# Identification of necroptosis & mitophagy-related key genes and their prognostic value in colorectal cancer

**DOI:** 10.1007/s12672-025-02221-y

**Published:** 2025-04-04

**Authors:** Xiuling Zhang, Li Meng, Tingjian Zu, Qian Zhou

**Affiliations:** 1 Department of Internal Medicine, The Hospital of Shandong Normal University, Jinan, 250014 Shandong China; 2 Department of Pharmacy, Weifang People’S Hospital, Shandong Second Medical University, Weifang, 261041 Shandong China; 3 School of Stomatology, Shandong First Medical University & Shandong Academy of Medical Sciences, Jinan, 250117 Shandong China; 4 Department of Pharmacy, Shandong Provincial Key Medical and Health Discipline of Clinical Pharmacy, Shandong Provincial Third Hospital, Shandong University, Jinan, 250013 Shandong China

**Keywords:** Colorectal cancer, Necroptosis, Mitophagy, UCHL1, HSPA1A, MAPK8, PLEC

## Abstract

**Background:**

Our study aimed to elucidate the potential necroptotic&mitophagy-related key genes in colorectal cancer (COAD) by bioinformatics analysis and identify their prognostic value in COAD.

**Methods:**

Firstly, we integrated the cancer genome atlas (TCGA) and gene expression omnibus (GEO) datasets to identify necroptosis & mitophagy-related differentially expressed genes (N&MRDEGs) in COAD using “TCGAbiolinks” and “GEOquery” packages. Secondly, the obtained data were used for differential expression analysis using the “limma” package, and further functional enrichment analysis using the “clusterProfiler” package. Then, gene set enrichment analysis (GSEA) and gene set variation analysis (GSVA) were utilized to explore pathway associations of the N&MRDEGs. Thirdly, the predictive model was developed utilizing LASSO (Least absolute shrinkage and selection regression) regression implemented through the “glmnet” package and validated via Kaplan–Meier analysis. Finally, we validated the function of the key genes by receiver operating characteristic (ROC) curve analysis, multivariate cox proportional hazards model and COAD cell lines.

**Results:**

There is a strong association between the 4 key genes (*UCHL1, HSPA1A, MAPK8,* and *PLEC*) of COAD and the necroptotic&mitophagy, which were found to be lowly mRNA level in COAD cell lines. Among them, *PLEC* exhibited a pronounced contribution to the utility of the model in the TCGA database and *UCHL1* has excellent diagnostic potential with an area under the curve (AUC) greater than 0.9.

**Conclusions:**

The perspective of bioinformatics analysis provides robust evidence suggested that *UCHL1*, *HSPA1A*, *MAPK8*, and *PLEC* genes are the prognostic biomarkers of COAD, the predictive model established herein provides a novel tool for risk stratification in clinical practice and serves as a foundation for further investigation into its underlying molecular mechanisms.

**Supplementary Information:**

The online version contains supplementary material available at 10.1007/s12672-025-02221-y.

## Introduction

Colorectal cancer (COAD) ranks third most common malignancy in developed countries, with a lethality rate of 48.9% in 2018 [1, 2]. Despite advancements in screening and treatment, the five-year survival rate remains around 65%, with the prognosis worsening dramatically once the disease progresses to metastatic stages [3]. The current therapeutic strategies, including surgery, chemotherapy, and radiotherapy, often fail at the molecular level targeting COAD, leading to drug resistance, recurrence, and metastasis [4–6]. This underscores the critical need for new prognostic biomarkers and drug targets to enhance patient outcomes.

Necroptosis and mitophagy, pivotal processes in cell death regulation and homeostasis, have recently been implicated in COAD pathogenesis [2, 7–10]. Necroptosis, a form of programmed cell death distinct from apoptosis, has been shown to play a dual role in cancer, acting as either a tumor suppressor or promoter depending on the cell context [11]. For example, Shikonin treatment has been shown to inhibit tumor growth in primary and metastatic osteosarcomas by enhancing tumor necrosis in vivo [12, 13], while in vitro studies demonstrate that endothelial cells undergo RIPK1/3-MLKL-dependent necroptosis when co-cultured with tumor cells, which facilitates tumor metastasis [14]. Mitophagy, a selective autophagic process essential for mitochondrial quality control, promotes cancer cell survival under stress conditions [15]. Both necroptosis and mitophagy have been implicated in COAD progression, emphasizing the necessity of identifying related differentially expressed genes (DEGs) to elucidate the underlying mechanisms [9, 16–20]. Furthermore, their roles in tumorigenesis and resistance in other cancers also underscore their potential as therapeutic targets and prognostic biomarkers in COAD [21, 22].

Our study utilized bioinformatics tools and publicly available datasets to elucidate the differences and functional roles of necroptosis&mitophagy-related differentially expressed genes (N&MRDEGs) in COAD, subsequently developing a prognostic model capable of stratifying patients based on risk and guiding personalized therapeutic strategies.

## Material and methods

### Data source

The COAD dataset was downloaded from the cancer genome project (TCGA, The Cancer Genome Atlas, https://www.tcgabiolinks) using the “TCGAbiolinks” package [23], containing 480 colon cancer samples and 41 healthy samples. Then, the count data were standardized in Fragments Per Kilobaseper Million (FPKM) format. The clinical data corresponding to samples were acquired from the UCSC database (http://genome.ucsc.edu) [24].

The expression profiles of the GSE41657 and GSE44076 datasets [25] were retrieved from the GEO database [26] using the “GEOquery” package [27], with GSE41657 comprising 25 colorectal cancer samples and 12 normal adjacent samples based on the GPL6480 Agilent-014850 whole human genome microarray, and GSE44076 including 98 colon cancer samples and 98 normal samples based on the GPL13667 platform, as detailed in Table 1. The above data was downloaded in November 2023. To ensure accurate gene annotation across platforms, probe IDs from GPL6480 and GPL13667 platforms were mapped to corresponding genes using the respective chip platform annotation files. Principal component analysis (PCA) was performed both before and after batch effect removal to assess its effectiveness. The combined datasets were integrated using the “sva” package [28], normalized with the “limma” package [29], and validated through PCA [30].

**Table 1:** Colon Cancer data set information list

	TCGA	GSE41657	GSE44076
Species	Homo sapiens	Homo sapiens	Homo sapiens
Platform	–	GPL6480	GPL13667
COAD group	480	25	98
Normal group	41	12	98
Reference	–	–	[43]

*TCGA* The cancer genome atlas, *COAD* Colorectal cancer, *GEO* Gene expression omnibus

647 necroptosis-related genes (NRGs) were obtained from the GeneCards database [31, 32] (https://www.genecards.org/) using “necroptosis” as the search keyword, with only protein-coding NRGs retained from eight necroptosis-related MSigDB database (https://www.gsea-msigdb.org/), as detailed in Table S1. Similarly, 538 mitophagy-related genes (MRGs) also were collected from the GeneCards database [32] by searching for “mitophagy” and retaining only protein-coding MRGs with “relevance score” > 1, as listed in Table S2. Necroptoses and mitophagy-related genes (N&MRGs) were obtained by the intersection of NRGs and MRGs (Table S3).

### N&MRDEGs identification

**Fig. 1:**
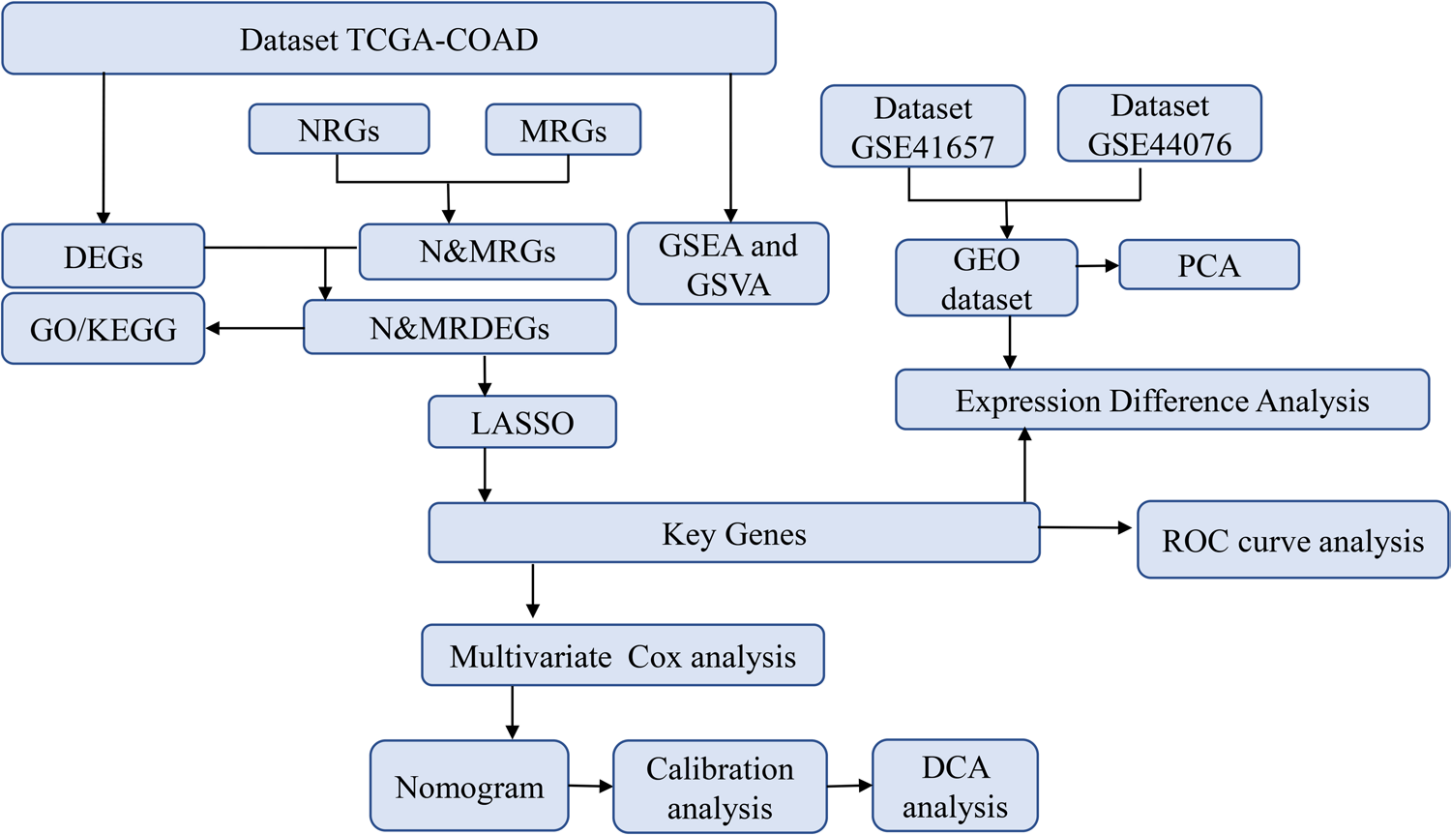
Flow chart of this study. TCGA, the cancer genome atlas; DEGs, differentially expressed genes; NRGs, Necroptosis-related genes; MRGs, Mitophagy-related genes; N&MRGs, Necroptoses and mitophagy-related genes; N&MRDEGs, Necroptosis and mitophagy-related differentially expressed genes; GO, Gene ontology; KEGG, Kyoto encyclopedia of genes and genomes; LASSO, least absolute shrinkage and selection regression; DCA, Decision curve analysis; ROC, Receiver operating characteristic

DEGs between the COAD group and the normal group, downloaded from the TCGA database, were analyzed using the “limma” package, with cutoff criteria set as |logFC|> 0.5 and adj. *p* < 0.05. Venn diagrams were created using the Venn Diagrams software to display the overlap of DEGs (defined as N&MRDEGs) between TCGA&DEGs and N&MRGs (Fig. 1).

### Functional enrichment analysis

To investigate the biological process and potential signaling pathways associated with necroptosis & mitophagy-related genes in the TCGA-COAD model, GO and KEGG analyses were performed using the “clusterprofiler” package [33], with statistical significance defined as *p* < 0.05 and FDR value (*q* value) < 0.25, applying the Benjamini-Hochberg (BH) for *p-value* correction.

### GSEA and GSVA

The TCGA-COAD genes are grouped according to the median log|FC| values and the enrichment analysis (GSEA) was performed using the “clusterprofiler” package. GSEA analysis parameters included a seed of 2020, 1000 computations, and the minimum and maximum number of genes were set as 10 and 500, respectively. The “c2.cp.all.v2022.1.Hs.symbols.gmt [All Canonical Pathways](3050)” gene set obtained from MSigDB database [34] was employed for GSEA, and the screening criteria of GSEA were set as *p* < 0.05 and *q* < 0.25 with the benjamini-hochberg (BH) method being used for p-value correction.

In addition to the differential expression analyses, the GSVA analysis was also conducted to enrich the different signal pathways in TCGA-COAD samples (COAD/Normal), for which the reference gene set “h.all.v7.4.symbols.gmt” was acquired from the MSigDB database and the screening criterion for significant enrichment was defined as p < 0.05; subsequently, the expression matrix was subjected to GSVA and the enriched pathways in the GSVA results were calculated.

### Construction of colorectal cancer prognostic model

To develop a prognostic model for colorectal cancer using the TCGA database, LASSO regression analysis was conducted on the expression levels of N&MRDEGs via the “glmnet” package^35^. Risk scores were calculated based on the penalty coefficients of each prognostic N&MRDEG, and COAD patients were stratified into high- and low-risk groups using the median risk score as the cutoff. Kaplan–Meier (KM) curve analysis, performed with the “survival” package, compared overall survival between the two groups, while the chromosomal locations of prognostic N&MRDEGs were visualized using the “RCircos” package [36].

$$\text{risk}Score = \sum_{i}Coefficient \left({hub\, gene}_{i}\right)*mRNA\, Expression\, (hub\, {gene}_{i})$$

### Friends analysis

The functional correlation of key genes was calculated using the “GOSemSim” package [37], and subsequently, this calculated correlation was analyzed via functional similarity analysis (Friends analysis).

### Differential expression analysis of key genes

The colorectal cancer-related datasets GSE41657 and GSE44076 were merged, after which batch effects were removed using the “sva” package, and normalization was carried out with the “limma” package to generate the combined dataset. Subsequently, differential expression analysis of key genes in the TCGA—COAD database and the combined dataset was performed by employing the Mann–Whitney U test, and the results were visualized via the “ggplot2” package. Key genes were then screened based on the identified expression differences. Finally, receiver operating characteristic (ROC) curves were plotted using the “pROC” package [38], and the area under the curve (AUC) was calculated to evaluate the diagnostic efficacy of N&MRDEGs in predicting the survival of COAD patients.

### Multivariate cox prognostic model building

To develop a multivariate Cox prognostic model for key genes in the TCGA database, univariate Cox regression analysis was first performed, retaining variables with p < 0.1 for inclusion. The “rms” package was then utilized to construct a nomogram [39], followed by the generation of a calibration curve [40] to assess the accuracy and discrimination of the multivariate Cox prognostic nomogram. Additionally, a decision curve analysis (DCA) plot was created using the “ggDCA” package to evaluate the predictive performance of the multivariate Cox prognostic model [41].

### Cell lines

LoVo (SCSP-514), SW480 (TCHu172), SW620 (TCHu101), and RKO (TCHu116) cell lines were obtained from the National Collection of Authenticated Cell Cultures, while CCD841 (CRL-1790) and HT29 (HTB-38) cell lines were sourced from ATCC (VA, USA), and HCT116 cell lines were maintained in our laboratory. LoVo cells were cultured in F-12 K medium (L450KJ, BasalMedia, China) supplemented with 10% fetal bovine serum (FBS; 04–001-1ACS, Biological Industries, Israel) and 1% penicillin/streptomycin (P/S), SW480 and SW620 cells in L-15 medium (L620KJ, BasalMedia, China) with 10% FBS and 1% P/S, RKO and CCD841cells in MEM medium (L510KJ, BasalMedia, China) with 10% FBS and 1% P/S and HT29 and HCT116 cells in DMEM medium (L110KJ, BasalMedia, China) with 10% FBS and 1% P/S.

### Real-time quantitative PCR (qPCR)

qPCR was performed as previously described [42], briefly, RNA was extracted with trizol (15596026CN, Thermo, USA) and reverse transcription was performed using the HiScript IV RT SuperMix agent (R423-01, Vazyme, China). Then, qPCR was carried out with SYBR kit (Q312-02, Vazyme, China) in QuantStudio 3 System (A28567, Applied Biosystems, United States). The sequences of the primers are shown as Table 2.

**Table 2:** Sequences of the primers

Gene	Sequence
UCHL1	Forward (F), 5′- GACGAATGCCTTTTCCGGTG-3′,
	Reverse (R), 5′- GAAGCGGACTTCTCCTTGCT-3′;
HSPA1A	Forward (F), 5′-TCAGAGGTCTACAGATGAAGGC-3′,
	Reverse (R), 5′-CCAGGGGCTATTGGCAAAGG-3′;
PLEC	Forward (F), 5′-GCCTTTCCAAGATTGCTGTT-3′,
	Reverse (R), 5′-TCAACATTGCAAACACAGGA-3′
MAPK8	Forward (F), 5′-TCTGGTATGATCCTTCTGAAGCA-3′,
	Reverse (R), 5′-TCCTCCAAGTCCATAACTTCCTT-3′
GAPDH	Forward (F), 5′-ACAACTTTGGTATCGTGGAAGG-3′,
	Reverse (R), 5′-GCCATCACGCCACAGTTTC-3′

### Statistical analysis

All data processing and analysis were conducted using R software (Version 4.1.2), with results expressed as mean ± standard deviation and analyzed in Prism 8.0 (GraphPad). Group comparisons were performed using the Wilcoxon rank sum test for two groups and the Kruskal–Wallis test for three or more groups, with statistical significance set at p < 0.05.

## Results

### COAD-associated necroptosis & mitophagy-related DEGs

**Fig. 2:**
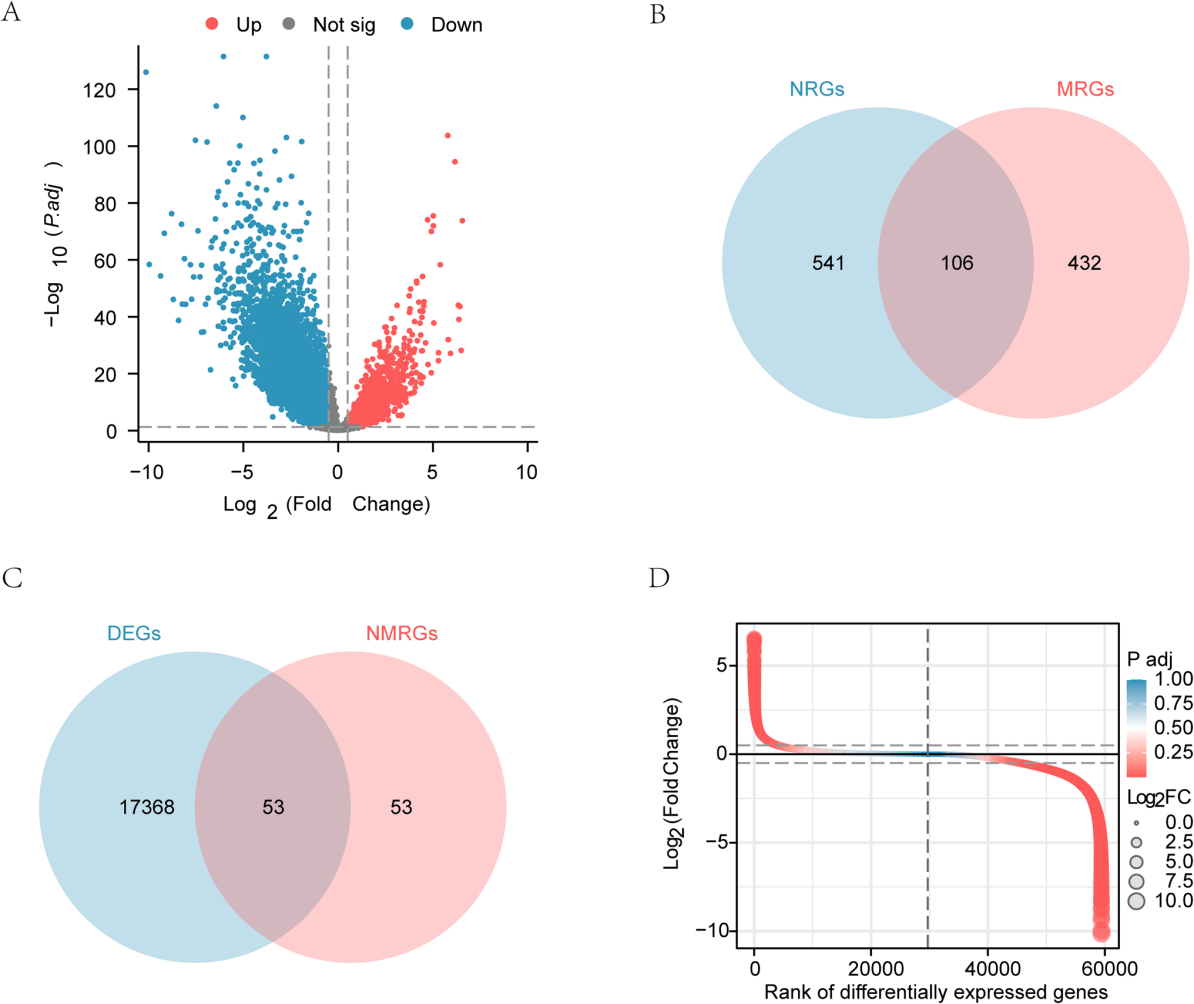
Differential Gene Expression Analysis. **A** Analysis of volcano plot of differentially expressed genes between COAD group and Normal group in MRGs dataset. **B** Venn diagram of NRGs and MRGs. **C** Venn diagram of DEGs in TCGA database and necroptosis & mitophagy related genes (N&MRGs). **D** Differential ranking map of N&MRDEGs in TCGA database. DEGs, differentially expressed genes; NRGs, Necroptosis-related genes; MRGs, Mitophagy-related genes; N&MRDEGs, Necroptosis&Mitophagy-related differentially expressed genes. COAD, Colorectal cancer

To analyze the DEGs in COAD and normal volunteers, we first downloaded the data from the TCGA database and obtained 480 COAD samples and 41 normal samples. The DEGs between the two groups were obtained by using the “limma” package (|logFC|> 0.5 and adj.* p* < 0.05), which included 3859 upregulated and 13,562 downregulated genes (Fig. 2A and Table S4).

To obtain the N&MRDEGs in COAD from TCGA, we first intersected 647 NRGs and 538 MRGs, yielding 106 necroptosis and mitophagy-related genes (N&MRGs), as illustrated in the Venn diagram (Fig. 2B). Subsequently, 53 N&MRDEGs were identified by intersecting the 17,421 DEGs obtained from COAD and normal samples (|logFC|> 0.5 and adj.* p* < 0.05) with the 106 N&MRGs, visualized in a Venn Diagram (Fig. 2C and Table S5), and the difference sort diagram was drawn with “pheatmap” R package (Fig. 2D).

### Gene ontology (GO) and pathway (KEGG) enrichment analysis of N&MRDEGs

**Fig. 3:**
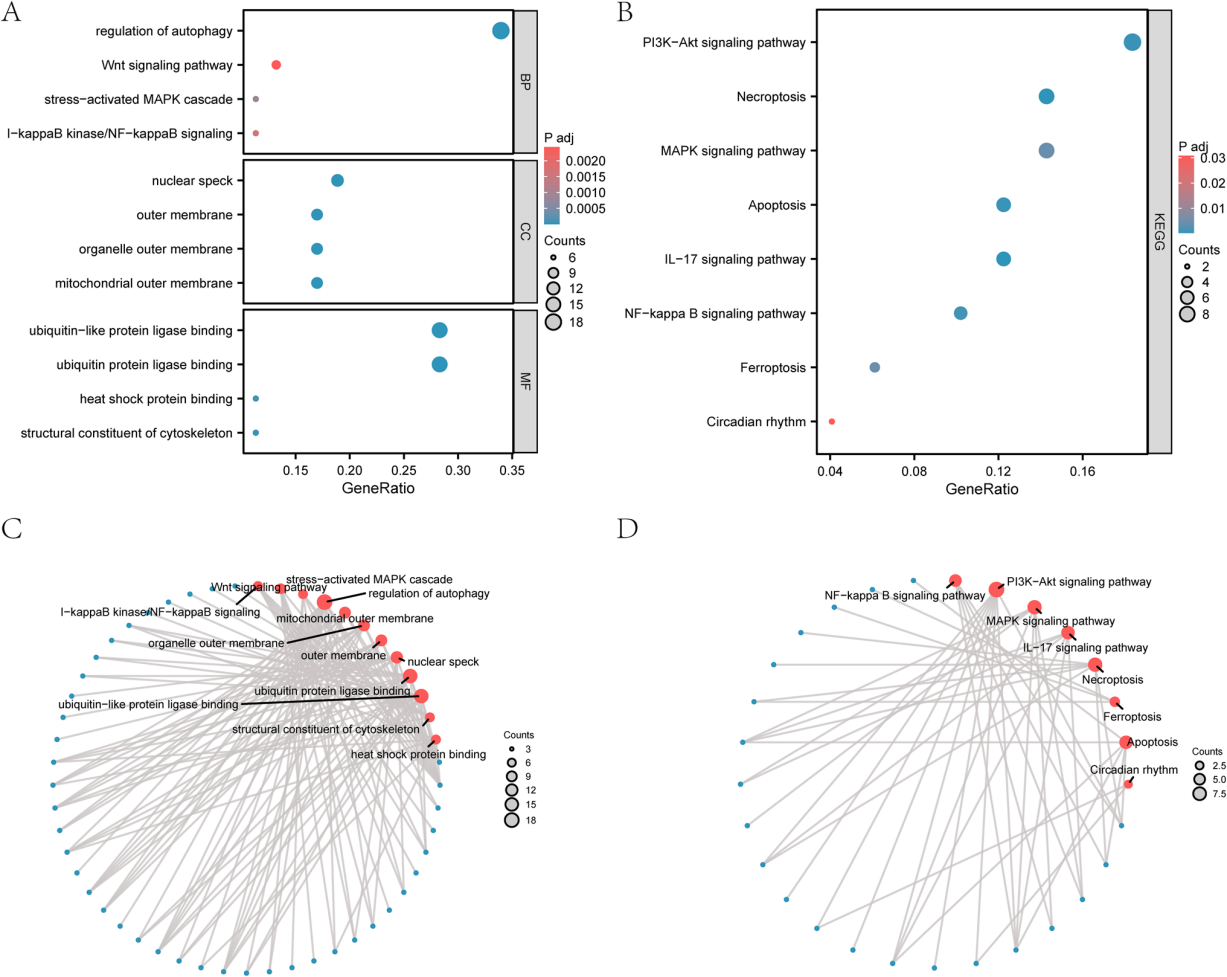
GO and KEGG Enrichment Analysis for N&MRDEGs. **A, B** GO and KEGG pathway enrichment analysis results of N&MRDEGs are presented in a bubble plot. GO terms and KEGG terms are shown on the abscissa. **C, D** GO and KEGG pathway enrichment analysis results of N&MRDEGs network. Red nodes represent items, blue nodes represent molecules, and the lines represent the relationship between items and molecules. The screening criteria for GO and KEGG enrichment analysis were adj. *p* < 0.05 and FDR value (*q* value) < 0.25, and the *p value* correction method was Benjamini-Hochberg (BH). N&MRDEGs, necroptosis & mitophagy-related differentially expressed genes; GO, Gene ontology; KEGG, Kyoto encyclopedia of genes and genomes; BP, Biological process; CC, Cellular component; MF, Molecular function

53 N&MRDEGs were obtained by the GO and KEGG enrichment analysis (Table 3) and visualized by bubble plots (Fig. 3A, B). The most enriched biological processes (BP) included the stress-activated MAPK cascade and regulation of autophagy, while cellular components (CC) comprised the organelle outer membrane, outer membrane, mitochondrial outer membrane and nuclear speck. Molecular functions (MF) were predominantly associated with ubiquitin protein ligase binding, structural constituent of the cytoskeleton, ubiquitin-like protein ligase binding and heat shock protein binding. KEGG analysis revealed that these DEGs were primarily involved in the NF-κB, PI3K-Akt, MAPK, and IL-17 signaling pathways, as well as necroptosis, ferroptosis, apoptosis, and circadian rhythm.

**Table 3:** GO/KEGG enrichment analysis for N&MRDEGs

Ontology	ID	Description	GeneRatio	BgRatio	P value	p.adjust
BP	GO:0010506	Regulation of autophagy	18/53	336/18800	8.05e−19	2.18e−15
BP	GO:0016236	Macroautophagy	17/53	306/18800	4.83e−18	6.54e−15
BP	GO:0016241	Regulation of macroautophagy	14/53	158/18800	8.85e−18	8e−15
BP	GO:0062197	Cellular response to chemical stress	17/53	332/18800	1.91e−17	1.29e−14
BP	GO:0000422	Autophagy of mitochondrion	11/53	88/18800	8.04e−16	3.63e−13
CC	GO:0005741	Mitochondrial outer membrane	9/53	205/19594	3.75e−09	8.74e−07
CC	GO:0031968	Organelle outer membrane	9/53	232/19594	1.1e−08	9.24e−07
CC	GO:0019867	Outer membrane	9/53	234/19594	1.19e−08	9.24e−07
CC	GO:0016607	Nuclear speck	10/53	411/19594	1.29e−07	7.51e−06
CC	GO:0005776	Autophagosome	6/53	112/19594	5.62e−07	2.62e−05
MF	GO:0031625	Ubiquitin protein ligase binding	15/53	298/18410	3.47e−15	9.55e−13
MF	GO:0044389	Ubiquitin-like protein ligase binding	15/53	317/18410	8.64e−15	1.19e−12
MF	GO:0005200	Structural constituent of cytoskeleton	6/53	104/18410	5.2e−07	4.77e−05
MF	GO:0031072	Heat shock protein binding	6/53	123/18410	1.4e−06	8.85e−05
MF	GO:0042826	Histone deacetylase binding	6/53	126/18410	1.61e−06	8.85e−05
KEGG	hsa04140	Autophagy—animal	15/49	141/8164	1.63e−15	3.06e−13
KEGG	hsa04137	Mitophagy—animal	12/49	72/8164	6.07e−15	5.7e−13
KEGG	hsa05131	Shigellosis	16/49	247/8164	4.17e−13	2.62e−11
KEGG	hsa05022	Pathways of neurodegeneration-multiple diseases	20/49	476/8164	8.13e−13	3.82e−11
KEGG	hsa04621	NOD-like receptor signaling pathway	13/49	184/8164	3.28e−11	1.23e−09

*GO* Gene ontology, *BP* Biological process, *CC* cellular component. *MF* Molecular function, *KEGG* Kyoto Encyclopedia of genes and genomes

Network diagrams of BP, CC, MF, and biological pathways were constructed based on GO/KEGG enrichment analysis (Fig. 3C, D), highlighting key processes and pathways such as nuclear speck, ubiquitin protein ligase binding, mitochondrial outer membrane, MAPK cascade, Wnt signaling, PI3K-Akt signaling, necroptosis, apoptosis, IL-17 signaling, NF-κB signaling, ferroptosis, and circadian rhythm.

### GSEA and GSVA analysis of N&MRDEGs

**Fig. 4:**
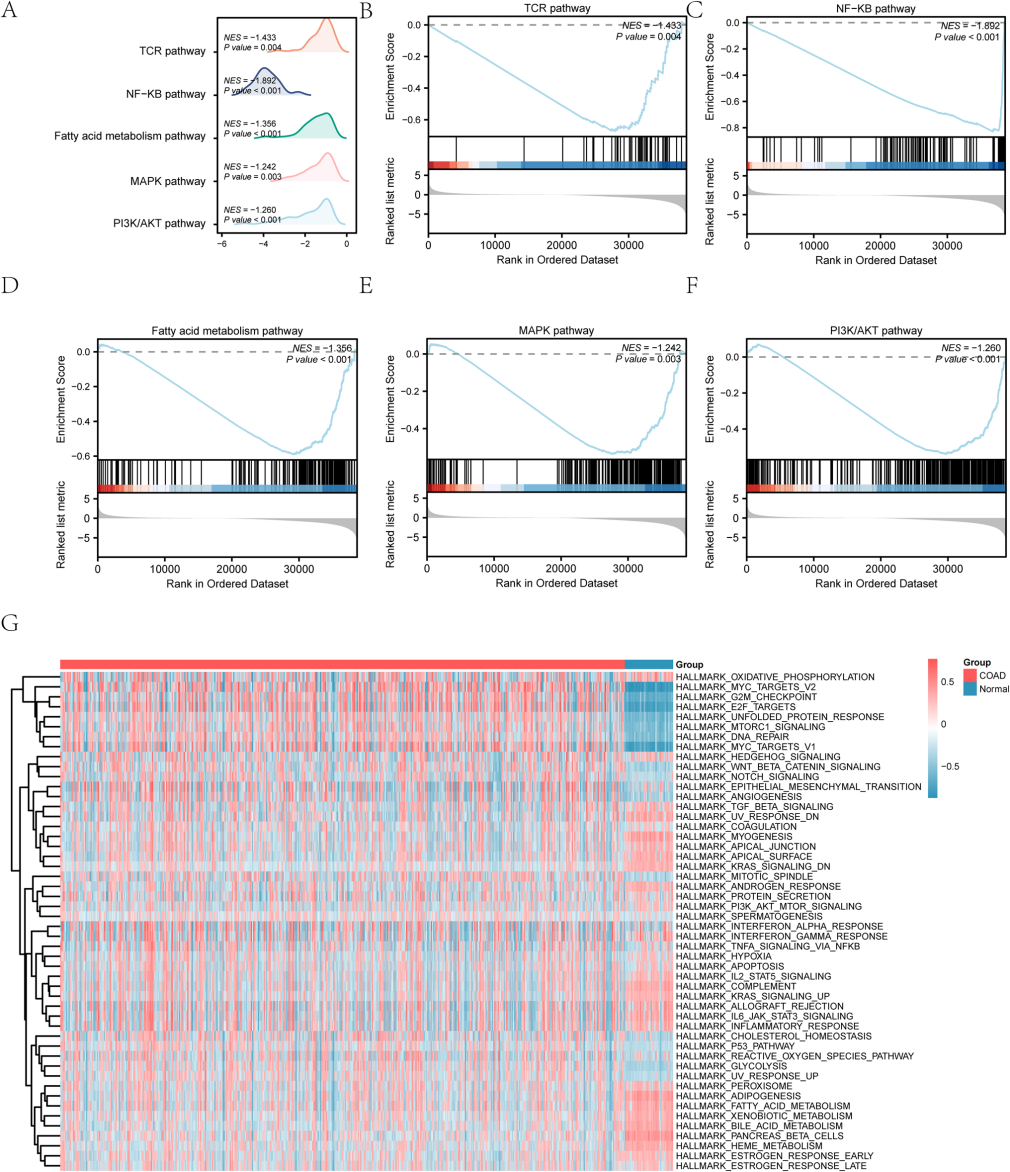
GSEA and GSVA analysis of TCGA database. **A** The mountain map of GSEA from the TCGA database. **B–F** GSEA analysis of N&MRDEGs in COAD. Represent the pathways about function enrichment of N&MRDEGs. **G** Heat map of GSVA results between COAD and Normal group in the TCGA dataset. *p* < 0.05, *p value* correction methods for Benjamini-Hochberg (BH). Red represents the COAD group and blue represents the Normal group. GSEA, Gene set enrichment analysis. N&MRDEGs, Necroptosis & mitophagy related differentially expressed genes; COAD, Colorectal cancer; GSVA, Gene set variation analysis

Next, the GSEA analysis of N&MRDEGs in COAD data revealed significant involvement in the TCR pathway, NF-κB pathway, fatty acid metabolism pathway, MAPK pathway, and PI3K/AKT pathway, among other biologically relevant functions and signaling pathways (Fig. 4A–F, Table 4).

**Table 4:** Results of GSEA for TCGA-COAD

ID	setSize	Enrichmen tScore	NES	*p* value	*p* adjust	*q* value
Reactome_CD22_Mediated_BCR_Regulation	61	−0.9655	−2.1196	1.00E−10	1.04E−08	8.42E−09
Reactome_Scavenging_of _Heme_From_Plasma	69	−0.9371	−2.0696	1.00E−10	1.04E−08	8.42E−09
Reactome_Fcgr_Activation	69	−0.9211	−2.0344	1.00E−10	1.04E−08	8.42E−09
Reactome_Role_of_Lat2_Ntal_Lab_on _Calcium_Mobilization	71	−0.9157	−2.0260	1.00E−10	1.04E−08	8.42E−09
Reactome_Creation_of_C4_and_C2_Activators	71	−0.9069	−2.0064	1.00E−10	1.04E−08	8.42E−09
Reactome_Binding_and_uptake_of_ligand_by_Scavenger _Receptors	98	−0.8756	−1.9655	1.00E−10	1.04E−08	8.42E−09
Reactome_Initial_Triggering_of_Complement	79	−0.8773	−1.9502	1.00E−10	1.04E−08	8.42E−09
Reactome_Fceri_Mediated_Mapk_Activation	87	−0.8682	−1.9439	1.00E−10	1.04E−08	8.42E−09
Reactome_Antigen_Activates_B_Cell_Receptor_BCR_Leading_to_Generation_of_Second _Messengers	86	−0.8695	−1.9403	1.00E−10	1.04E−08	8.42E−09
Reactome_Fceri_Mediated_CA_2_Mobilization	86	−0.8599	−1.9188	1.00E−10	1.04E−08	8.42E−09

*GSEA *Gene set enrichment analysis. *TCGA *The cancer genome atlas. *COAD *Colorectal cancer. *N&MRDEGs* Necroptosis and Mitophagy-related differentially expressed genes

To further explore the difference of the “h.all.v7.4.symbols.gmt” gene set between the COAD and the normal groups of the TCGA database, GSVA was performed, identifying statistically significant differences (p < 0.05) in fatty acid metabolism, TGFβ signaling, PI3K/AKT/mTOR signaling, hedgehog signaling, and oxidative phosphorylation (Table 5). Finally, the differential expression between the COAD and the normal groups was analyzed and visualized by heat map based on the results of GSVA (Fig. 4G).

**Table 5:** Results of GSVA for TCGA-COAD

Signalings logFC	logFC	AveExpr	t	*p*.Value	adj.*p*.Val
Hallmark_TGFβ_Signaling	0.2056	−0.0395	3.4826	0.0005	0.0007
Hallmark_PI3K/AKT/MTOR_Signaling	0.1352	−0.0493	2.5958	0.0097	0.0124
Hallmark_Fatty_Acid_Metabolism	0.3386	−0.0033	6.1048	1.95E−09	4.87E−09
Hallmark_Hedgehog_Signaling	0.1429	−0.0315	2.30089	0.0218	0.0266
Hallmark_Oxidative_Phosphorylation	0.2262	−0.0055	3.1501	0.0017	0.0023
Hallmark_Pancreas β_Cells	0.4907	−0.0182	10.1491	2.70E−22	1.93E−21
Hallmark_Kras_Signaling_DN	0.3920	−0.04525	9.4032	1.43E−19	8.93E−19
Hallmark_Bile_Acid_Metabolism	0.4211	−0.0435	8.8758	9.83E−18	4.92E−17
Hallmark_Heme_Metabolism	0.4067	−0.0363	8.81012	1.65E−17	7.48E−17
Hallmark_Myogenesis	0.4381	−0.0399	8.1413	2.64E−15	1.08E−14

*GSVA* Gene set variation analysis, *TCGA* The cancer genome atlas, *COAD *Colorectal cancer, *N&MRDEGs* Necroptosis & Mitophagy-related differentially expressed genes

### Construction of prognostic model for COAD

**Fig. 5:**
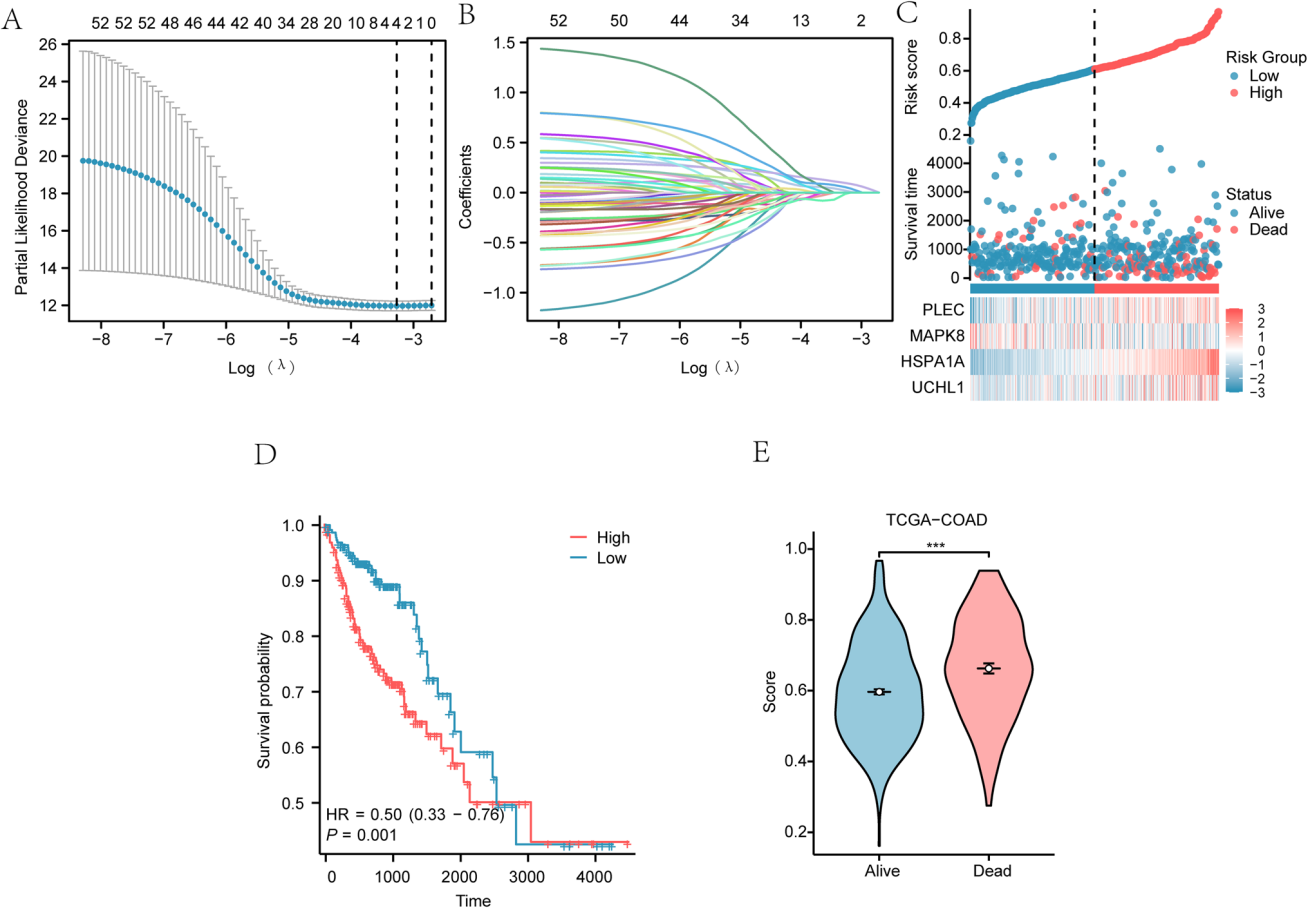
Construction of prognostic model for colorectal cancer. **A，B** Prognostic model plot of LASSO regression model (**A**) and variable trajectory plot (**B**). **C** The LASSO regression model of risk factors. The risk factor diagram is composed of three parts: Risk grouping, Survival outcomes and Heatmap. **D**, **E** Prognostic KM curve (**D**) and group comparison graph (**E**) between RiskScore of LASSO regression model and OS survival outcome of TCGA database patients. ***p < 0.001. TCGA, The cancer genome atlas. COAD, Colorectal cancer. LASSO, least absolute shrinkage and selection regression; KM, Kaplan–Meier; OS, Overall survival

To assess the prognostic value of the 53 N&MRDEGs in the TCGA database, LASSO regression analysis was conducted, with results visualized with a LASSO regression model plot (Fig. 5A) and a LASSO variable trajectory plot (Fig. 5B). Four genes including *UCHL1*, *HSPA1A*, *MAPK8*, *and PLEC*, were identified as the key genes and served as key diagnostic markers for colon cancer. To further validate the prognostic value of the four N&MRDEGs in the TCGA databases, the sample grouping in the constructed prognostic model of colorectal cancer was visualized by a risk factor map (Fig. 5C). Subsequently, we performed a prognostic Kaplan–Meier curve analysis based on the LASSO risk score (Risk Score) combined with the overall survival (OS) of COAD in the TCGA database (Fig. 5D). The results revealed that the risk scores of the LASSO regression model had a highly significant difference in predicting the survival outcome (OS) of patients (*p* < 0.05), and the prognosis of patients in the High-risk group (High) was worse. Furthermore, a grouping comparison chart integrating patient prognosis and COAD information revealed highly significant differences in RiskScore among different OS outcome groups (*p* < 0.001) (Fig. 5E).

### The key genes in the expressions of the LASSO regression model between high and low-risk score variance analysis

**Fig. 6:**
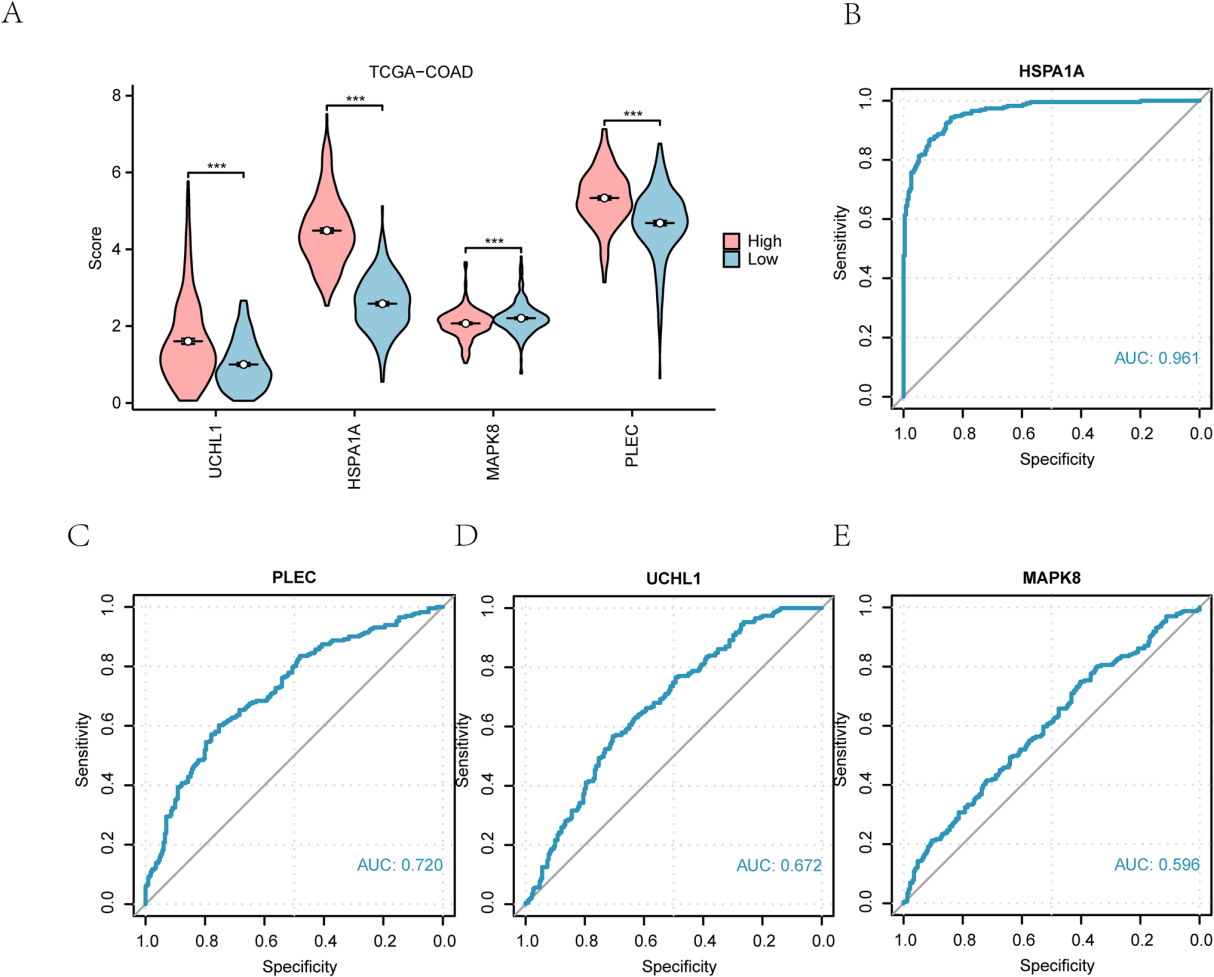
Expression difference analysis of key genes between high and low RiskScore groups in LASSO regression model. **A** Group comparison chart results of key genes between high and low RiskScore groups in LASSO regression model are shown. Blue represents the low RiskScore group and red represents the high RiskScore group, ***p < 0.001. **B**–**E** ROC curve analysis of key genes including HSPA1A (**B**), PLEC (**C**), UCHL1 (**D**), MAPK8 (**E**) in LASSO regression model between high and low RiskScore groups. The closer the AUC in the ROC curve is to 1, the better the diagnostic effect is. The AUC had a certain accuracy in the range of 0.7–0.9. AUC between 0.5 and 0.7 had a low accuracy. LASSO, least absolute shrinkage and selection regression; ROC, Receiver operating characteristic; AUC, Area under curve

The relationship between the four key genes (*UCHL1*, *HSPA1A*, *MAPK8*, *PLEC*) and the risk score group (High/Low) in the LASSO regression model was analyzed (Fig. 6A), revealing significantly higher expression levels of these genes in the high-risk group compared to the low-risk group (p < 0.001).

Then, the ROC curves for the four key genes were generated to evaluate their predictive accuracy between risk score groups (Fig. 6B–E), which showed that the UCHL1 and MAPK8 exhibited moderate accuracy (0.5 < AUC < 0.7), while HSPA1A and PLEC demonstrated higher accuracy (0.7 < AUC < 0.9) in distinguishing between high- and low-risk groups.

### Differential expression analysis of key genes between COAD and normal groups in TCGA and GEO database

**Fig. 7:**
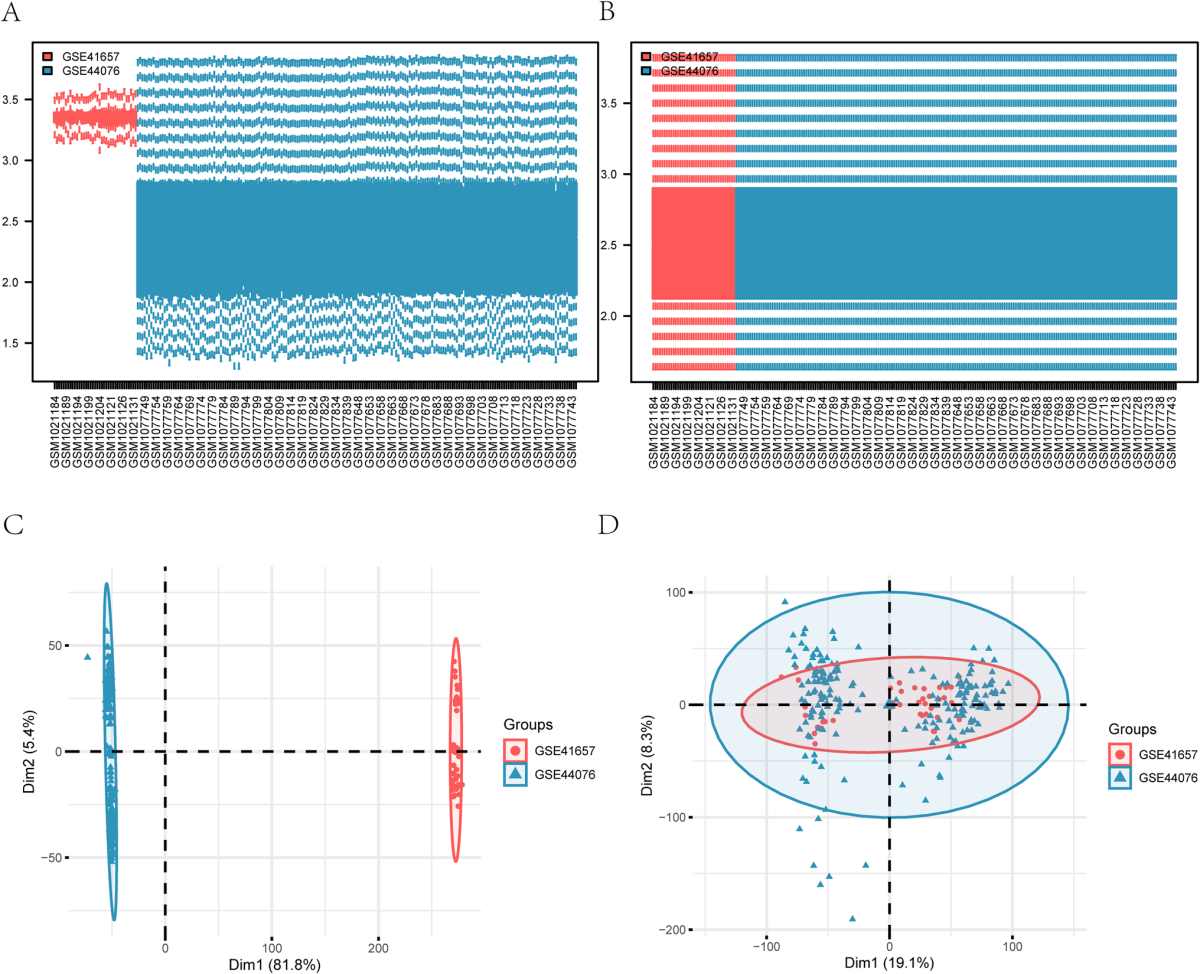
Debauching of the dataset. **A**, **B** Boxplot plots of combined datasets before (**A**) and after (**B**) normalization. **C**, **D** PCA plots of combined datasets before (**C**) and after (**D**) batch effect removal processing. Red represents the GSE41657 dataset, blue represents the GSE44076 dataset. PCA, Principal component analysis

To begin with, the “sva” package was utilized to acquire the integrated GEO database derived from GSE41657 and GSE44076. During this process, the batch effect inherent in the two datasets was effectively eliminated (Fig. 7A-B). Subsequently, the PCA plot was generated, which clearly demonstrated that the batch effect of samples within the COAD dataset had been successfully eradicated after batch removal (Fig. 7C, D).

**Fig. 8:**
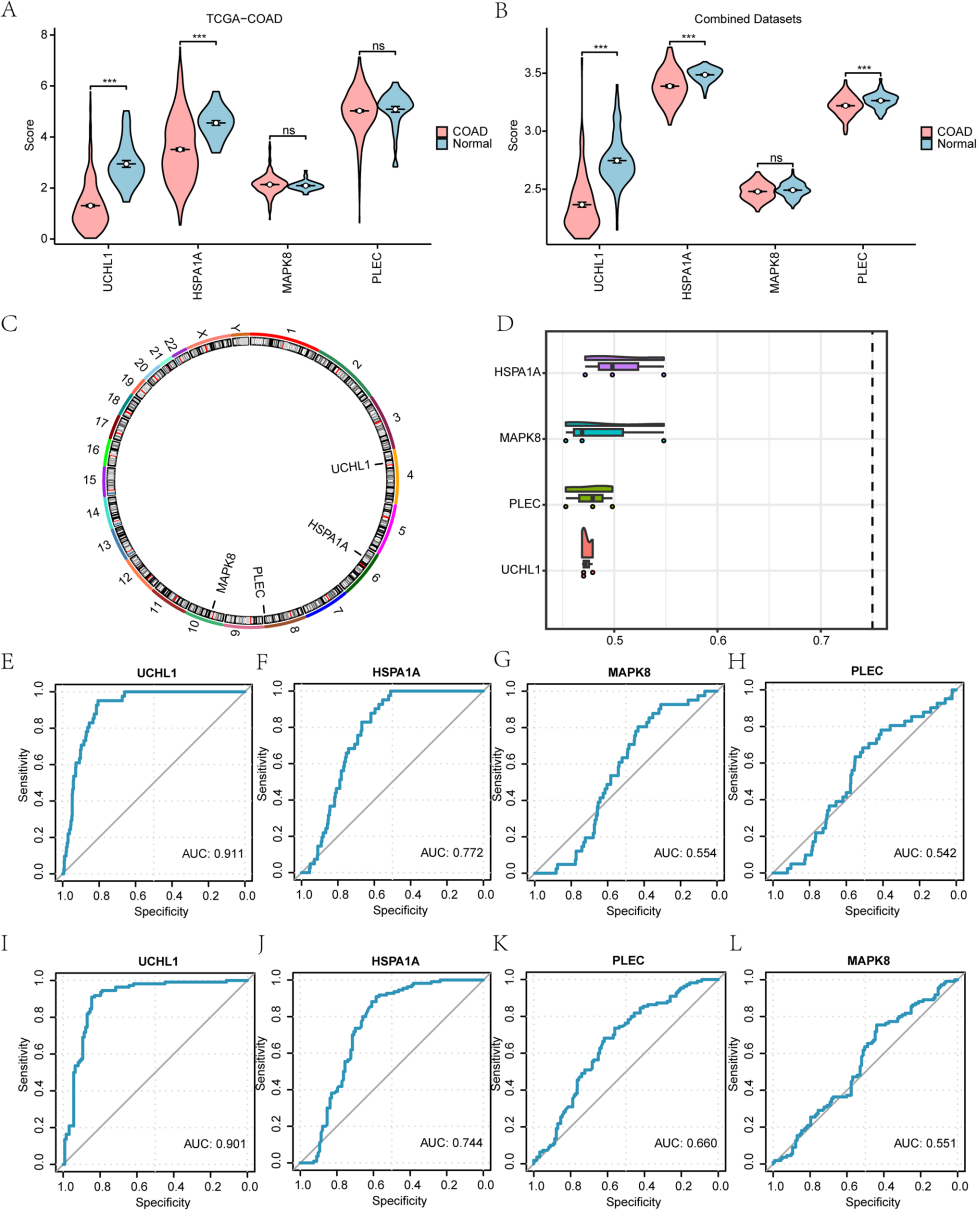
Differential expression analysis of key genes between cancer group and normal group in TCGA and combined datasets. **A, B** Plot of group comparison of key genes between Normal and COAD groups in TCGA database (**A**) and combined datasets (**B**). **C** Mapping of key genes in human chromosomes. **D** Cloud rain map of key genes in Friends analysis. **E–L** ROC curve analysis of key genes including HSPA1A, PLEC, UCHL1 and MAPK8 in TCGA (**E**–**H**) and Combined Dataset (**I**–**L**). ***p < 0.001. TCGA, The cancer genome atlas; ROC, Receiver operating characteristic

Next, we further identified the expression of four key genes (*UCHL1*, *HSPA1A*, *MAPK8*, *PLEC*) in COAD using the TCGA and combined GEO datasets, revealed significantly decreased expression of UCHL1 and HSPA1A in the COAD group compared to normal samples (Fig. 8A, B), Chromosomal localization analysis via the “RCircos” package mapped UCHL1 to chromosome 4, HSPA1A to chromosome 6, PLEC to chromosome 8, and MAPK8 to chromosome 10 (Fig. 8C).

Friends’ analysis identified HSPA1A as a critical gene in COAD biological processes, being closest to the critical value (Fig. 8D). Finally, ROC curve analysis demonstrated high accuracy for *UCHL1* in both TCGA (AUC > 0.9) (Fig. 8E) and combined GEO datasets (AUC > 0.9) (Fig. 8I), while HSPA1A showed moderate accuracy (0.7 < AUC < 0.9) in both datasets (Fig. 8F, J). MAPK8 and PLEC exhibited low accuracy (0.5 < AUC < 0.7) in TCGA (Fig. 8G, H) and combined GEO datasets (Fig. 8K, L).

### TCGA datasets COAD multivariable cox prognosis model building

**Fig. 9:**
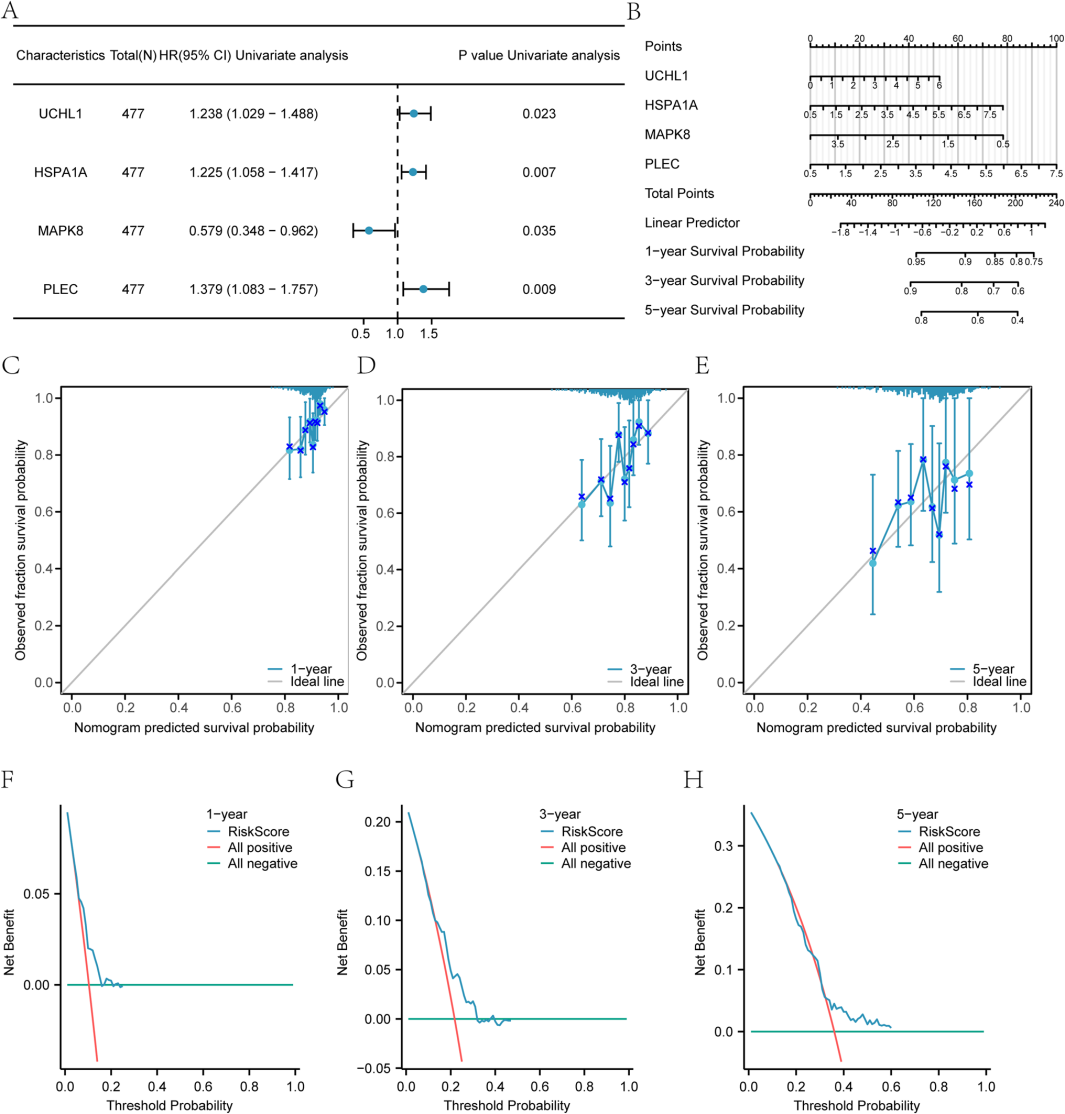
Construction of multivariate Cox regression model in TCGA database. **A** Forest plot of the univariate Cox regression model of the TCGA database. **B** Nomogram of multi-factors Cox regression model. **C–E** Calibration curves at 1 year (**C**), 3 years (**D**), and 5 years (**E**) for multivariate Cox regression model nomogram analysis. **F–H** DCA plots at 1 year (**F**), 3 years (**G**), and 5 years (**H**) of the multivariate Cox regression model. DCA, Decision Curve Analysis; TCGA, The cancer genome atlas

To evaluate the prognostic significance of four key genes (*UCHL1, HSPA1A, MAPK8* and *PLEC*) in the TCGA database, clinical data from non-duplicated COAD patients were statistically analyzed (Table 6). Univariate Cox regression identified genes meeting p < 0.1 for inclusion in multivariate Cox regression, constructing a prognostic model (Fig. 9A). A nomogram, integrating variables from the multivariate model via weighted scoring, demonstrated PLEC as the most influential prognostic variable (Fig. 9B, Table S6). Calibration analysis of the nomogram revealed superior predictive accuracy for 1-year survival compared to 3- and 5-year outcomes (Fig. 9C–E). Decision curve analysis (DCA) further validated the model’s clinical utility, with predictive efficacy ranked as 3-year > 5-year > 1-year (Fig. 9F–H).

**Table 6:** Results of Cox Analysis

Characteristics	Total (N)	Univariate analysis		Multivariate analysis	
		Hazard ratio (95% CI)	*p *value	Hazard ratio (95% CI)	*p *value
UCHL1	477	1.238 (1.029–1.488)	0.023	1.141 (0.938–1.386)	0.1864
HSPA1A	477	1.225 (1.058–1.417)	0.007	1.170 (1.000–1.369)	0.0498
MAPK8	477	0.579 (0.348–0.962)	0.035	0.714 (0.414–1.233)	0.2271
PLEC	477	1.379 (1.083–1.757)	0.009	1.240 (0.954–1.612)	0.1083

N&MRDEGs: Necroptosis and Mitophagy-related differentially expressed genes. OS: Overall survival. TCGA: The cancer genome atlas. COAD: Colorectal cancer

### Validation of key genes associated with necroptosis and mitophagy in COAD cells

**Fig. 10:**
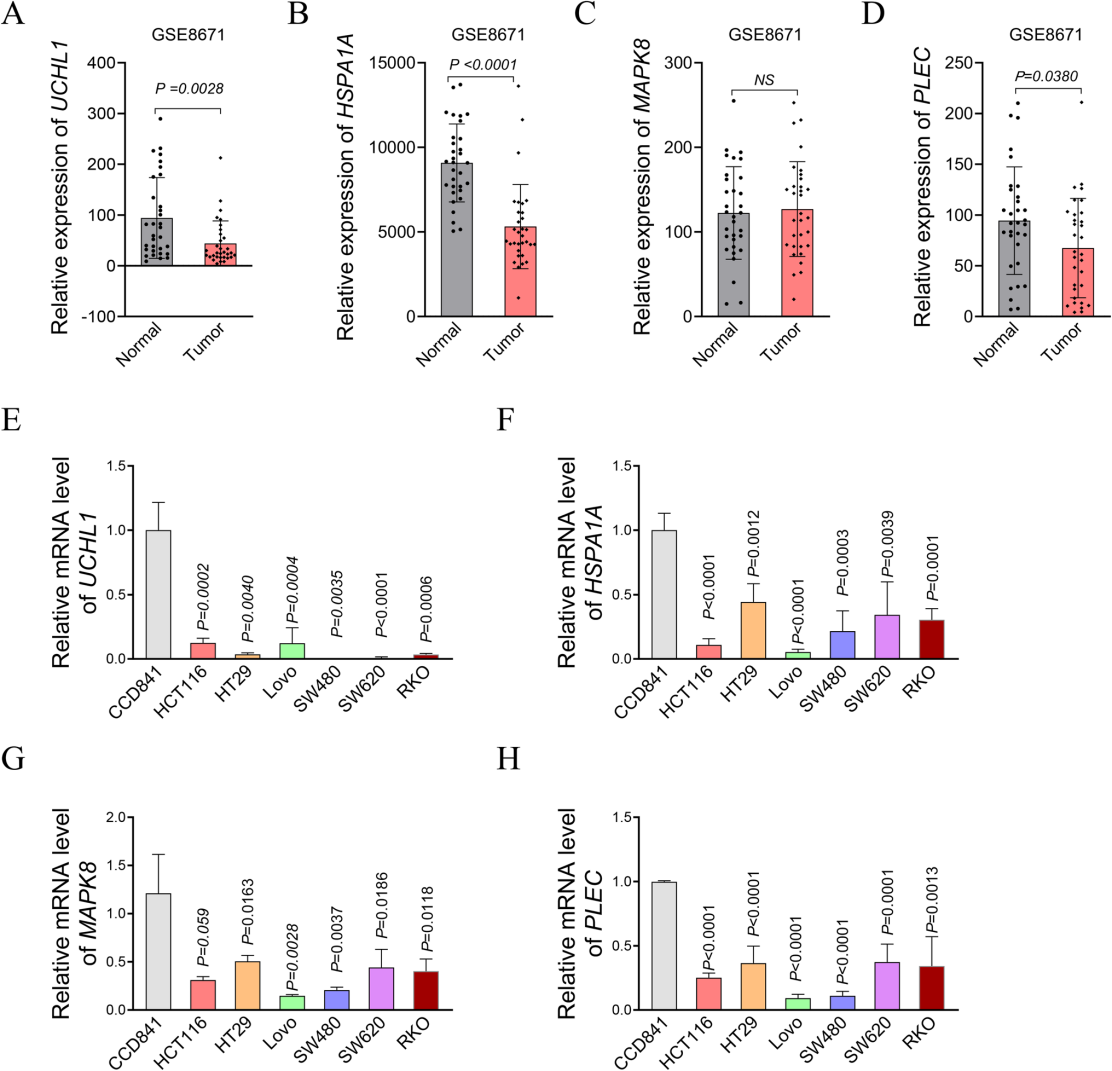
Validation of the four key N&MRDEGs in COAD cells. **A–D** The mRNA levels of key N&MRDEGs in COAD cells based on GSE8671 analysis. **E–H** Quantitative PCR analysis of key N&MRDEGs mRNA level in human normal colonic epithelial cells and COAD cell lines. Data were presented as mean ± SD from three independent experiments

To further validate the expression of the four key N&MRDEGs, we examined the mRNA levels of four key N&MRDEGs using GSE8671 dataset, mRNA levels were assessed using the GSE8671 dataset, revealing significantly lower expression of UCHL1, HSPA1A, and PLEC in colorectal adenoma compared to normal colonic mucosal tissue, while MAPK8 showed comparable expression between groups (Fig. 10A–D), consistent with prior findings in Fig. 8B. Additionally, we also detected the mRNA level of the four key N&MRDEGs in COAD cell lines. Compared with the CCD841 cells, a human normal colonic epithelial cell, all the four key N&MRDEGs showed a marked decrease consistently in COAD cell lines (Fig. 10E–H).

## Discussion

Our study identifies *UCHL1, HSPA1A, MAPK8,* and *PLEC* as the prognostic biomarkers for COAD, with the constructed prognostic model offering a novel tool for clinical risk stratification and insights into underlying molecular mechanisms. We validated a set of necroptosis&mitophagy-related differentially expressed genes (N&MRDEGs) in COAD, highlighting their roles in cell death and energy metabolism, processes critical to tumor pathogenesis. Functional enrichment analysis revealed their involvement in pathways essential for cellular homeostasis and stress responses, often dysregulated in cancer[44]. The LASSO regression-based prognostic model, incorporating UCHL1, HSPA1A, MAPK8, and PLEC, underscores their potential as survival predictors in COAD.

PLEC, a cytolinker protein, emerged as a significant prognostic factor[45], likely influencing tumor cell mechanical strength, adhesion, migration, and invasion[46], while also mediating drug resistance, though its precise mechanisms require further investigation [47]. UCHL1, a deubiquitinating enzyme, plays a critical role in protein degradation and turnover, with its aberrant expression linked to tumor progression and metastasis [48]. Its high diagnostic accuracy (AUC > 0.9) in distinguishing COAD from normal tissue underscores its potential as a diagnostic marker. Additionally, UCHL1’s involvement in cell cycle regulation and apoptosis [49] suggests its downregulation in COAD may promote tumor cell proliferation and survival by impairing responses to harmful signals (such as DNA damage), making tumor cells more active in proliferation and more likely to survive.

Despite this study having provided valuable insights into the expression differences and functional associations of necroptosis&mitophagy-related genes in COAD, it has limitations remaining, including the absence of wet-lab validation, a potentially limited sample size for capturing genetic variability, and insufficient clinical validation, lacking larger multi-center datasets validation, Batch effects, though mitigated, may persist in multi-dataset analyses. Future research should integrate experimental and larger multi-center datasets validation, expand sample sizes, and enhance clinical validation to strengthen translational applicability.

In conclusion, our findings provide a comprehensive analysis of necroptosis&mitophagy-related DEGs in COAD, offering a prognostic model with clinical relevance and identifying key genes (including *UCHL1, HSPA1A, MAPK8,* and *PLEC*) as potential therapeutic targets and diagnostic markers. These results advance our understanding of COAD pathogenesis and pave the way for improved patient management strategies.

## Supplementary Information

Additional file 1.

Additional file 2.

Additional file 3.

Additional file 4.

Additional file 5.

Additional file 6.

## Data Availability

The datasets generated during and/or analyzed during the current study are available from the corresponding author upon reasonable request. Code availability The codes used and/or analyzed during the current study are available from the corresponding author on reasonable request.

## Code availability

The codes used and/or analyzed during the current study are available from the corresponding author on reasonable request.
